# History with Heart—and Impact: The National Library of Medicine Michael E. DeBakey Fellowship in the History of Medicine

**DOI:** 10.14797/mdcvj.1047

**Published:** 2021-12-15

**Authors:** Jeffrey S. Reznick, Kenneth M. Koyle

**Affiliations:** 1 National Library of Medicine, National Institutes of Health, Bethesda, Maryland, US

**Keywords:** research, history, humanities, public service

## Abstract

The National Library of Medicine (NLM) Michael E. DeBakey Fellowship in the History of Medicine is a unique program in a unique institution: the world’s largest biomedical library, which is part of the National Institutes of Health (NIH). The fellowship is rooted in strong connections between its namesake and the NLM and is an expression of Dr. Michael E. DeBakey’s longstanding appreciation of the library and the role of the humanities in medical education and practice. This article explains Dr. DeBakey’s connections to the NLM and describes the origins, development, and structure of the NLM Michael E. DeBakey Fellowship in the History of Medicine. It also highlights research achievements of selected NLM Michael E. DeBakey Fellows, demonstrating that the fellowship is successfully carrying forward Dr. DeBakey’s principles and practices of weaving science, technology, and the humanities to form holistic understanding of the human condition, inspiring well-rounded careers built on both scientific and humanistic knowledge.

## Michael E. DeBakey and the NLM

During his 75-year career, Dr. Michael E. DeBakey (1908-2008) transformed cardiovascular surgery, raised medical education standards, and shaped national healthcare policy. He pioneered dozens of operative procedures such as aneurysm repair, coronary bypass, and endarterectomy, which routinely save thousands of lives each year, and performed some of the first heart transplants. Dr. DeBakey’s inventions included the roller pump (a key component of heart-lung machines) as well as artificial hearts and ventricular assist pumps. He was a driving force in building Houston’s Baylor University College of Medicine into a premier medical center, where he trained several generations of top surgeons from all over the world.

Dr. DeBakey played a central role in the establishment of the National Library of Medicine (NLM). He was a visionary supporter of the institution, serving two terms as chairman of its Board of Regents and actively participating in many of its programs, from the establishment of the National Network of Libraries of Medicine in the 1960s to launching NLM outreach initiatives in the 1990s, and promoting the digitization of its indexes to pre-1960s journal articles.

**Figure 1:**
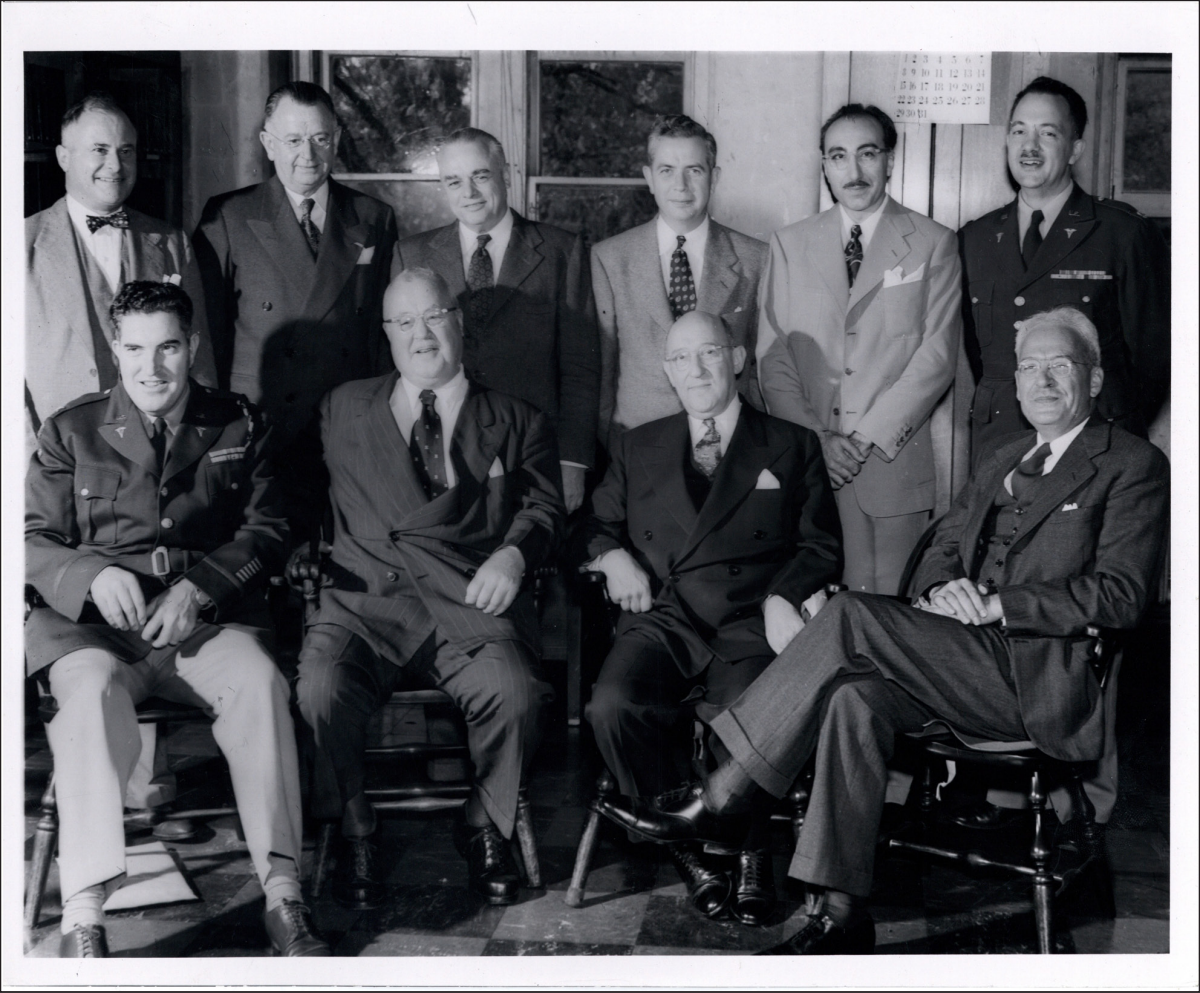
Michael E. DeBakey, MD, standing, second from right, among the honorary consultants to the Army Medical Library, 1950. Courtesy National Library of Medicine, National Institutes of Health.

Dr. DeBakey valued a well-rounded approach to medicine. During World War II, when he was serving as a surgical consultant to the Army Surgeon General’s Office in Washington, DC, he spent countless hours in the Army Medical Library (AML), gaining an appreciation for its collections as well as its tenuous and fraught circumstances as an underfunded, often neglected part of the Army Medical Department. After the war, Dr. DeBakey was appointed as an honorary consultant to the AML, and, in this capacity, he presented a detailed analysis of the library’s status (***Figure 1***).

Dr. DeBakey argued that the AML had outgrown its original mandate to serve the US Army and had developed into a national institution with a civilian function, that it was expected to fulfill this expanded role without proper authorization, and finally, that the military could not justify the funding of civilian functions, but no other legislation provided funding either. The purpose and function of the library as the National Library of Medicine, as Dr. DeBakey saw it, needed to be defined and authorized by new legislation.

In 1955, as part of the second Hoover Commission Medical Task Force, Dr. DeBakey encouraged senators Lister Hill (an Alabama Democrat and the son of a prominent surgeon) and John F. Kennedy (Democrat from Massachusetts) to draft legislation for a National Library of Medicine as part of the US Public Health Service. The senators introduced the bill in March 1956; with a push from Dr. DeBakey, it made it to the senate floor for a vote, and the National Library of Medicine Act was signed into law by President Eisenhower on August 3, 1956.

**Figure 2:**
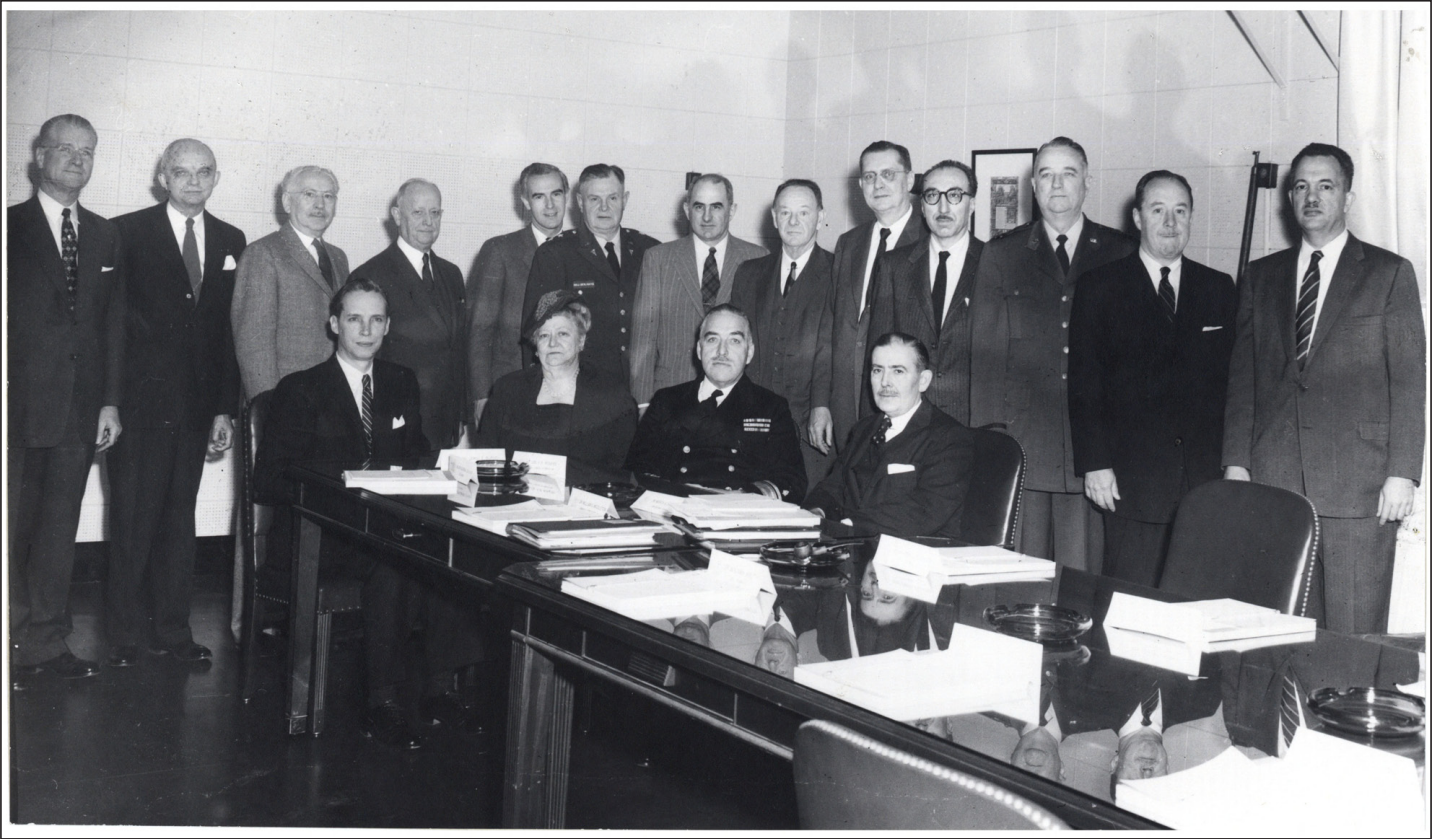
Michael E. DeBakey, MD, standing, fourth from right, at the first meeting of the National Library of Medicine’s Board of Regents, during which one of the primary agenda items was determining a new permanent location of the library. Courtesy National Library of Medicine, National Institutes of Health.

The first NLM Board of Regents met in March 1957, with Dr. DeBakey as an appointed member (***Figure 2***). One of the first orders of business was establishing a home for the new library, a challenge Dr. DeBakey took on with enthusiasm. The pros and cons of about 10 locations were discussed, and as part of the following meeting one month later on April 29, most of the regents went on a bus tour of several of the locations.

DeBakey campaigned for the new building to be located on the campus of the National Institutes of Health in Bethesda, Maryland. “I felt strongly that the Library had to be related to a substantive medical scientific activity,” DeBakey later recalled. “Research nourishes the Library; it is an indispensable resource before the investigator begins the research, and it provides new information on completion of the research—a cycle that repeats itself continuously.”^1^

**Figure 3:**
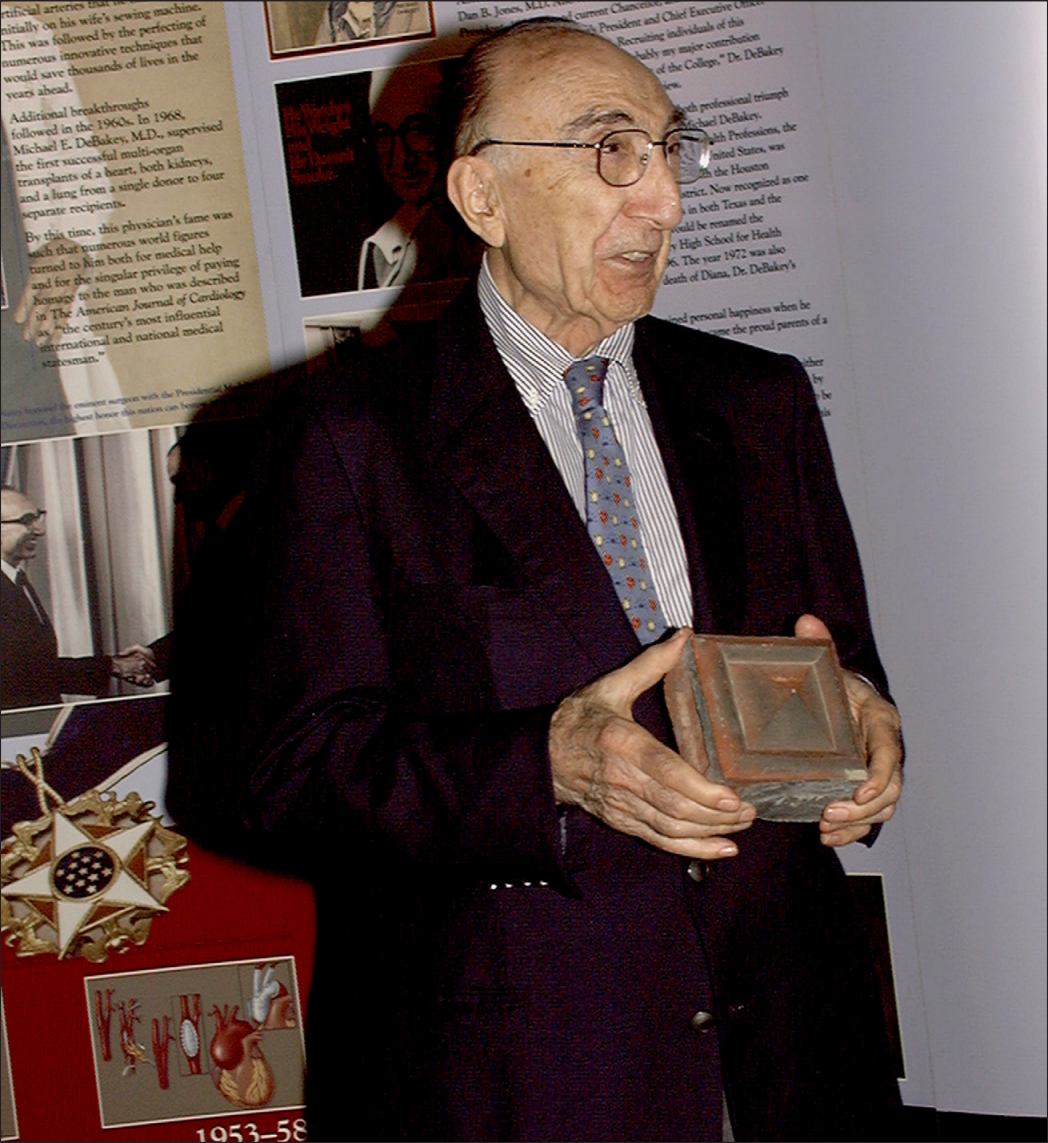
Michael E. DeBakey, MD, on the occasion of his 90th birthday in 1998, holding an object that represented his solid and enduring appreciation of the library: a brick from its previous headquarters on Independence Avenue in Washington, DC. Courtesy National Library of Medicine, National Institutes of Health.

After much debate, the Board agreed with Dr. DeBakey’s proposal and selected the NIH campus as the home of the new NLM. In 1959, the Board of Regents Chairman appointed Dr. DeBakey to serve on a working group to consult about the design of the new building, and less than 3 years later, in January 1962, the NLM opened to the public. Dr. DeBakey continued to serve on the board for many years, in two separate tenures. A few years later, expressing this appreciation of the NLM and other medical libraries, Dr. DeBakey stated: “Our libraries are our most valuable and precise link between generations and between people working in many areas. We must protect them at any cost.”^2^ Dr. DeBakey’s appreciation of the NLM and its collections never waned (***Figure 3***).

## The NLM Michael E. DeBakey Fellowship

In February 2016, the NLM received a generous gift from The DeBakey Medical Foundation to support development, expansion, and enhanced public access to the library’s collection of Michael E. DeBakey papers and to develop related programs in the history of medicine, specifically the NLM Michael E. DeBakey Fellowship in the History of Medicine. The gift also supported free videocasting and permanent archiving of NLM History Talks so they could be widely appreciated.^3^ Five years on, these programs have successfully carried forward, preserved, and made widely known the dynamic legacy of Michael E. DeBakey.

Launched in April 2016, the NLM Michael E. DeBakey Fellowship in the History of Medicine provides selected individuals with up to $10,000 to support onsite research in the collections of the NLM, which span ten centuries, encompass a variety of digital and physical formats, and originate from nearly every part of the globe.^4^ The collections include papers of Michael E. DeBakey himself, reflecting the expanse of his life, achievements, and interests as a world-renowned medical statesman, innovator, and champion of humanitarianism and life-long learning.

The fellowship is administered on behalf of the NLM by the Foundation for Advanced Education in the Sciences (FAES). Through this partnership, the NLM and FAES operate the fellowship on an annual cycle with calls for applications each spring, application deadlines each fall, and announcements of fellows each winter. FAES receives applications to the fellowship program through a dedicated online portal. Staff of the NLM History of Medicine Division and the Office of NIH History review submitted applications based primarily on the viability of the research plan, including its scholarly rigor and extent to which it engages NLM’s collections, as well as the feasibility of the plan given the proposed budget. Fellowships are awarded to individual applicants, not to institutions, to help offset the costs associated with visiting and using the NLM historical collections. Anyone over the age of 18, of any academic discipline and status, who has not previously received the Fellowship, is eligible. Non-US citizens may apply. On average, the NLM has named four fellows annually. Fellows are expected to make at least one in-person visit to the NLM to undertake their historical research projects during the calendar year following their selection. Fellows are also required to author at least one guest blog post, based on their research, for the popular blog of the NLM History of Medicine Division, Circulating Now. Understandably, the COVID-19 pandemic has impacted the fellowship, and the NLM expects flexibility in scheduling future visits of fellows when it reopens to support onsite research. In the interim, library staff are available to provide reference support and planning for onsite research.

## Publishing Achievements of NLM DeBakey Fellows

**Figure 4:**
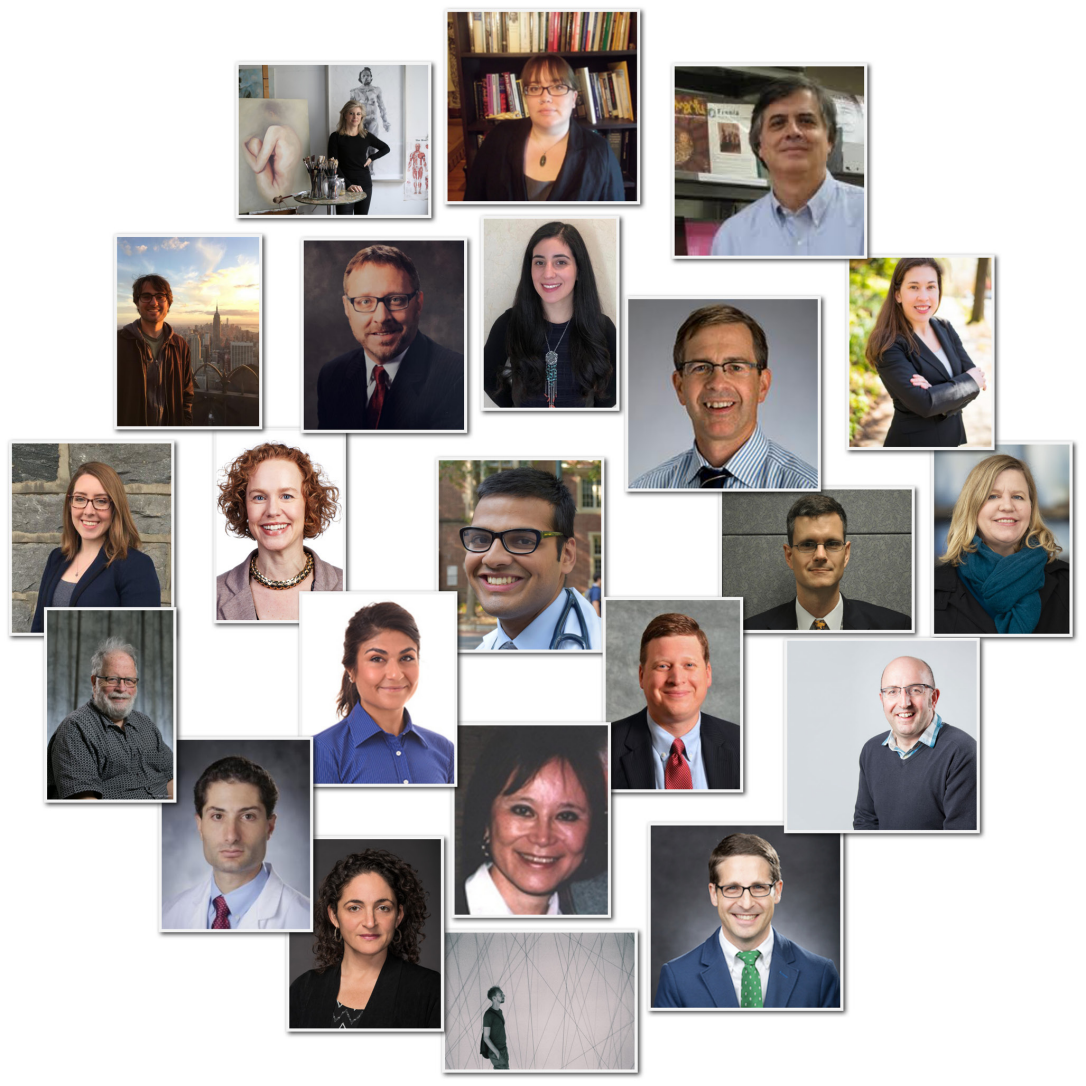
2016-2021 National Library of Medicine Michael E DeBakey Fellows in the History of Medicine. Courtesy National Library of Medicine, National Institutes of Health.

Since the launch of the NLM Michael E. DeBakey Fellowship in 2016, nearly two dozen individuals have been named to the fellowship (***Figure 4***). This international and multidisciplinary cohort of women and men at various stages of their careers has produced an impressive and measurable body of research work encompassing five scholarly books, four articles, and 16 essays on *Circulating Now*, which have been collectively viewed over 25,000 times.^5^

NLM DeBakey Fellows have published on a diversity of subjects ranging from the evolution of modern surgery to the development of medical libraries and medical librarianship to the interplay between health care and urban economies. They also have produced a global history of civilian internment during wartime, the first biography of Dr. DeBakey himself along with various detailed studies of his surgical techniques and innovations, a history of the internationalization of medical education, and a study of how doctors’ wives transformed American medicine and the evolution of wartime bacteriology and wound care.

The library presents an annual NLM Michael E. DeBakey Lecture in the History of Medicine, which complements the NLM Michael E. DeBakey Fellowship in the History of Medicine and further expands public awareness of Dr. DeBakey and his appreciation of well-rounded careers built on both scientific and humanistic understanding. Delivered by a selected fellow, the NLM DeBakey Lecture is a cornerstone of the NLM History Talks program, which promotes awareness and use of NLM and other historical collections for research, education, and public service in biomedicine, the social sciences, and the humanities. All NLM History Talks are livestreamed globally by NIH Videocasting, closed-captioned, permanently archived, and freely available at https://videocast.nih.gov/PastEvents?c=221. Notably, beginning in 2021, all NLM History Talks incorporate American Sign Language interpretation. Support for this expanded access to NLM History Talks is made possible in part through a gift to the NLM from the DeBakey Medical Foundation.

Together, the first five annual NLM Michael E. DeBakey Lectures, from 2017 through 2021, have been viewed more than 1,500 times, and their associated posts on *Circulating Now* have been read more than 6,500 times. This data, along with that associated with the posts authored by NLM DeBakey Fellows themselves, offers a quantitative measure by which these initiatives overall have raised public awareness of the name and legacy of Dr. DeBakey.

## Conclusion

The NLM Michael E. DeBakey Fellowship in the History of Medicine and its related initiatives are carrying forward Dr. DeBakey’s principles and practices of weaving science, technology, and the humanities to form a holistic understanding of the human condition, inspiring an ever-growing cohort of scholars to embrace his perspective by pursing well-rounded careers built on both scientific and humanistic knowledge. In stewarding this program and the legacy it represents, and no less the papers of Michael E. DeBakey himself, which document his life and career, the NLM is advancing the study of history with heart—and impact.
